# Bile acids and gut microbiota: interactions and impacts on polycystic ovary syndrome

**DOI:** 10.3389/frmbi.2025.1702198

**Published:** 2026-01-12

**Authors:** Haiqing Qian, Jialing Tao, Lingli Shi, Haiyan Sun, Li Yang, Li juan Cui, Wenting Xu, Lihong Wang

**Affiliations:** 1 Department of Reproduction, Zhangjiagang TCM Hospital Affiliated to Nanjing University of Chinese Medicine, Suzhou, Jiangsu, China; 2 Translational Medical Innovation Center, Zhangjiagang TCM Hospital Affiliated to Nanjing University of Chinese Medicine, Suzhou, Jiangsu, China; 3 Department of Anesthesiology, Zhangjiagang TCM Hospital Affiliated to Nanjing University of Chinese Medicine, Suzhou, Jiangsu, China; 4 Department of Gynaecology, Zhangjiagang TCM Hospital Affiliated to Nanjing University of Chinese Medicine, Suzhou, Jiangsu, China; 5 Department of Pathology, Zhangjiagang TCM Hospital Affiliated to Nanjing University of Chinese Medicine, Suzhou, Jiangsu, China

**Keywords:** bile acids, gut microbiota, inflammation, intestinal epithelial barrier, metabolic homeostasis, polycystic ovary syndrome, sexual hormones

## Abstract

Polycystic ovary syndrome (PCOS) is a multifaceted endocrine and metabolic disorder intricately associated with hyperandrogenism (HA), insulin resistance (IR), chronic inflammation, and obesity. The gut microbiota (GM) is considered a mature endocrine organ capable of exerting multiple effects by regulating bile acids (BAs) metabolism. Disruption of GM homeostasis can initiate various pathological processes, including metabolic disorders, endocrine imbalances, low-grade inflammation, and reduced insulin sensitivity, thereby providing novel avenues for research into the pathogenesis of PCOS. There is bidirectional signalling between the GM and BAs: the microbial community tightly regulates the metabolism and synthesis of BAs, while the BAs pool and its composition affect the diversity and homeostasis of intestinal microorganisms. Dysregulation of BAs metabolism mediated by the GM may constitute a crucial pathological link in the progression of PCOS. The objective of this review is to investigate the function of BAs as a signalling molecule bridging the GM and PCOS, to synthesise the current understanding of the roles of BAs and intestinal microorganisms in the pathogenesis of PCOS, and to explore new treatment strategies for PCOS further.

## 1 Introduction

The gut microbiota is the most extensive microecosystem in the human body and plays a crucial role in maintaining host physiology (Fan and Pedersen, 2021). Often referred to as the “second genome,” it is acquired early in life and co-evolves with the host, influencing and enhancing physiological processes in a mutually beneficial manner (Bäckhed et al., 2005). This symbiotic relationship facilitates nutrient absorption, substance metabolism, immune regulation, and the maintenance of biological barriers (Clemente et al., 2012; Lozupone et al., 2012). Alterations in GM composition, known as dysbiosis, have been implicated in the onset of various physiological disorders. These include low-grade inflammation, metabolic dysfunctions, lipid accumulation, and reduced insulin sensitivity, all of which are intricately linked to the pathogenesis and progression of chronic metabolic diseases (Boulangé et al., 2016; Chassaing et al., 2016). The GM exerts a significant influence on the female reproductive endocrine system, affecting various stages of reproduction, including follicular and oocyte maturation in the ovaries, fertilisation, embryo migration, implantation, and the entire gestational process (Xu et al., 2023; Huang et al., 2024). It has been hypothesised that dysbiosis of the GM is associated with the onset and progression of polycystic ovary syndrome (PCOS) (Qi et al., 2019; Giampaolino et al., 2021; He and Li, 2021). Recent multi-omics studies have combined gut metagenomics with serum metabolomics to reveal unique microbial and metabolic characteristics associated with PCOS. These findings offer a comprehensive systems-level insight into the GM-host metabolic axis within the context of this syndrome (Yang et al., 2022; Li et al., 2025).

Bile is a digestive secretion that plays a crucial role in emulsifying and dissolving lipids, with bile acids (BAs) comprising approximately 50% of its organic composition (Li and Chiang, 2014). The biosynthesis of primary BAs predominantly occurs in hepatocytes within the liver. Upon entering the gastrointestinal tract, GM transform these primary BAs into over 50 distinct secondary BAs, each possessing unique chemical properties (Funabashi et al., 2020). Recent findings indicate that BAs exhibit pleiotropic effects, functioning as hormones or signalling molecules that regulate metabolism (Vítek and Haluzík, 2016; Cai et al., 2022). Their chemical diversity is attributed to the synergistic interactions between the host and GM (Winston and Theriot, 2020). The gut microbial community can be conceptualised as a mature “endocrine organ,” capable of influencing host physiology by producing BAs metabolites distinct from those synthesised in the liver (Ridlon and Bajaj, 2015; Chen et al., 2024). Nuclear receptors activated by BAs, including the farnesoid X receptor (FXR), pregnane X receptor (PXR), constitutive androstane receptor (CAR), vitamin D receptor (VDR), and G protein-coupled bile acid receptor (TGR5), play pivotal roles in regulating lipid, glucose, and energy metabolism, as well as in modulating inflammation, drug metabolism and detoxification processes (Lefebvre et al., 2009; Fuchs and Trauner, 2022). The size and composition of the BAs pool are intrinsically linked to the structure and distribution of the microbial community. It is plausible that specific microbial assemblages may lead to distinct variations in BAs profiles (Jia et al., 2020; Foley et al., 2021).

PCOS is a complex gynaecological endocrine disorder characterised by highly heterogeneous clinical manifestations, primarily including androgen excess, ovulatory dysfunction, and the presence of polycystic ovaries (Li et al., 2019; Stener-Victorin et al., 2020). Research indicates that elevated levels of conjugated primary BAs in the circulation of women with PCOS are positively associated with hyperandrogenism (HA) (Yu et al., 2023; Zhu et al., 2024). Furthermore, the BAs metabolic pathway is critically influenced by GM alterations observed in individuals with PCOS (Qi et al., 2019). A metagenomic analysis revealed that in the GM of patients with PCOS, the abundance of certain pathogenic bacteria, such as *Parabacteroides merdae* and *Bacteroides fragilis*, increased. On the contrary, the levels of beneficial microorganisms, such as *Faecalibacterium prausnitzii*, decreased significantly. Discovery can provide a basis for taxonomy and support the existence of imbalanced GM in PCOS (Chu et al., 2020). Consequently, GM regulates BAs metabolism to construct potential therapeutic targets for PCOS (Zhao et al., 2020; Zhang et al., 2022, 2023). It is crucial that the metabolic characteristics of patients and differences between lean and obese PCOS bring great diversity to understanding the pathogenesis of the disease. Obesity, as a key confounding factor, can independently drive changes in transcription factor composition, BAs metabolism, and systemic inflammation (Teede et al., 2023). Recent evidence shows that the lean type of PCOS exhibits different microbial metabolic characteristics compared to the obese type, which has differential effects on IR, HA, and chronic low-grade inflammation (Torres et al., 2018; Yu et al., 2023). Clinical studies systematically analysing the association between BA changes and the PCOS phenotype have summarised important experimental results (**Table 1**). Therefore, a nuanced discussion of the GM-BAs axis in PCOS must explicitly account for the syndrome’s metabolic heterogeneity. In this review, we will examine the potential modulatory or confounding effects of adiposity on the observed associations within the GM-BAs-PCOS axis. Although prior reviews have independently investigated the relationship between GM and PCOS, as well as the role of BAs in metabolic diseases, this review distinctively focuses on the GM-BAs axis as a pivotal mechanistic link connecting dysbiosis to the pathogenesis of PCOS. It also emphasises the therapeutic potential of modulating BAs receptors, such as FXR and TGR5, in the management of PCOS. This integrative perspective has not yet been comprehensively addressed in the literature. This review examines the latest evidence on the role of BAs as crucial signalling molecules mediating interactions between the GM and PCOS. Furthermore, it seeks to comprehensively integrate the role of BAs with the GM’s influence on the onset and progression of PCOS.

**Table 1:** Comprehensive summary of human studies investigating BAs and clinical correlations in PCOS.

Study (Year)	Design & population	Diagnostic criteria	BMI stratification	Major alterations in BAs profile	Key associations with PCOS phenotypes	Proposed mechanisms/pathways	Notable limitations
Cai et al. (2019)	RCT secondary analysis n=1000	Modified Rotterdam	Stratified (metabolic syndrome vs. HA)	↓ TBAs in metabolic syndrome↔ TBAs in HA ↓ TBAs in clomiphene treatment	↓ TBAs in metabolic syndrome ↑ Liver enzymes in HA	- Metabolic abnormalities: inflammation→↓ insulin signaling→altered liver function - HA: direct harmful to androgens on liver	- Secondary analysis - Exclusion severe liver dysfunction - Pregnancy may affect liver parameters
Jia et al. (2019)	Cross-sectional case-control 10 PCOS vs.10 controls	Rotterdam	No difference	↓ GCA	Linked to dyslipidemia (↑TG, TC, ↓HDL) Linked to HA and ↑ LH, LH/FSH , E2	- BAs dysregulation→dyslipidemia - Lipid metabolism disorder→IR and T_2_DM - ↑ DHEAS reflect adrenal contribution to HA	- Small sample size - Cross-sectional (not causality) - No confounders adjustment - Lack of validation cohort
Zhang et al. (2019)	Cross-sectional case-control 37 PCOS vs. 35 controls	Modified Rotterdam	No difference	↑ TBAs, GCA, GCDCA, TCA, TCDCA ↔ CA, CDCA ↔ Secondary BAs	Positively correlated with T and androstenedione	- BAs act via FXR in ovarian→inhibit aromatase→↓ androgen-to-E_2_ conversion - BAs promote inflammation/intestinal permeability→PCOS pathogenesis	- Small sample size - Cross-sectional (not causality) - Uncontrolled diet - Strict exclusion criteria (limited generalizability)
Qi et al. (2019)	Case-control 50 PCOS vs. 43 controls	Rotterdam criteria	No difference	↓ GDCA, TUDCA in stool/serum Negatively correlated with *B. vulgatus*	Negatively correlated with *B. vulgatus* Linked to ↓ IL-22, IR, ovarian dysfunction	- Gut microbiota (*B vulgatus*)→↓ GDCA/TUDCA→↓ IL-22→IR/ovarian dysfunction	- Limited to Chinese population - Causal needs validation
Yang et al. (2021)	Cross-sectional case-control 35 PCOS vs.31 controls (undergoing IVF/ICS)	Rotterdam criteria	No difference	↑ GCA, TCA, GCDCA, CDCA ↑ Total primary BAs, ↑ conjugated BAs	↑ GCDCA with FSH and LH ↑ CDCA with AFC No correlation with IR	- FXR signaling in granulosa cells - BAs in follicular→ovarian microenvironment→follicular dysfunction/anovulation	- IVF/ICSI cohort - Small sample size - Cross-sectional (not causality) - No mechanistic validation
Yang et al. (2022)	Case-control 32 PCOS vs. 18 controls	Rotterdam	Not stratified (PCOS BMI higher)	↑ TCA, TDCA in obese PCOS ↔ other BAs	Proinflammatory microbes ↑T, FAI, LH, AMH, HOMA-IR Beneficial microbes depleted with metabolic disturbances	- Gut dysbiosis→SREBF1/mTOR→PI3K/AKT→IR/HA/ovarian dysfunction	- Causality inferred from rat FMT - Uncontrolled diet/lifestyle - Untargeted metabolomics
Yoost et al. (2022)	Cross-sectional 27 young women (lean/obese ± PCOS)	Clinical HA + anovulation	Stratified (lean vs. obese) at a threshold of 30 kg/m²	↑ TCA, TDCA ↔ TBAs, glycine-conjugated Bas ↔ CA, CDCA	↑ FGF-21 in PCOS (regardless of obesity) FGF-21 correlated with TCA/T in obese PCOS ↔ FGF-19	- TCA stimulate FGF-21 in hepatocytes - Obese+PCOS amplify BAs-FGF21-androgen	- Small sample size - Unstandardized blood draws - Rural/high-obesity population - Cross-sectional (not causality)
Yu et al. (2023)	Cross-sectional 408 PCOS vs. 204 controls	Rotterdam	No difference	↑ Primary & secondary unconjugated Bas ↑ CDCA, LCA ↓ CA, DCA, GDCA, GLCA, TLCA ,GCDCA	DCA linked to insulin indices (influenced by T) HA-PCOS: ↑ CA, CDCA CDCA+LCA+T have diagnostic value for PCOS	- BAs act via FXR/TGR5 signaling - Gut microbiota-BAs-host signaling	- Cross- sectional (not causality) - BAs- gut flora relationship needs exploration - RCT needed for BAs-based treatment
Zhu et al. (2024)	Cross-sectional case-control 240 lean PCOS vs. 80 controls	Rotterdam	Lean only (BMI 18-25 kg/m²)	↑ Non-12-OH BAs, CDCA ↓ CA, DCA, GDCA ↓ CA/CDCA, 12-OH BAs/non-12-OH BAs	CDCA positively linked with T, FAI CA positively linked with T, DHEAS, FBG, HOMA-IR CA/CDCA positively correlated with DHEAS ↓ CA/CDCA as a compensatory response to↑ DHEAS	- Altered BAs as compensatory for HA - Androgen-regulated liver enzyme activity in BAs synthesis - Shift from classical to alternative BAs synthesis pathway (CYP8B1↓, CYP7B1↑)	- Cross- sectional (not causality) - Limited to lean women - BAs synthesis- HA mechanisms need validation

↑ indicate increase, ↓ indicate decrease; ↔ indicate no difference; AFC, antral follicle count; AKT, serine-threonine kinase; AMH, anti-Mullerian hormone; BAs, bile acids; BMI, body mass index; CA, cholic acid; CDCA, chenodeoxycholic acid; CYP7B1, cytochrome P450 7B1; CYP8B1, cytochrome P450 8B1; DCA, deoxycholic acid; DHEAS, dehydroepiandrosterone sulphate; E2, oestradiol; FAI, free androgen index; FBG, fasting blood glucose; FGF−19, fibroblast growth factor 19; FGF−21, fibroblast growth factor 21; FMT, fecal microbiota transplantation; FSH, follicle-stimulating hormone; FXR, farnesoid X receptor; GCA, glycocholic acid; GCDCA, glycochenodeoxycholic acid; GDCA, glycodeoxycholic acid; GLCA, glycolithocholic acid; HA, hyperandrogenism; HDL, high-density lipoprotein; HOMA-IR, homeostasis model assessment-estimated insulin resistance; IL-22, interleukin-22; IR, insulin resistance; LCA, lithocholic acid; LH, luteinizing hormone; mTOR, mammalian target of rapamycin; PI3K, phosphoinositide 3-kinase; RCT, randomised controlled trial; SREBF 1, sterol regulatory element binding transcription factor 1; T, testosterone; T2DM, type 2 diabetes mellitus; TBAs, total bile acids; TC, total cholesterol; TCA, taurocholic acid; TCDCA, taurochenodeoxycholic acid; TDCA, taurodeoxycholic acid; TG, triacylglycero; TGR5, G protein-coupled bile acid receptor; TLCA, taurolithocholic acid; TUDCA, tauroursodeoxycholic acid.

To systematically capture and synthesise evidence supporting the GM-BAs axis as a central mechanistic bridge in PCOS, we conducted a structured literature search in the Web of Science and PubMed databases, covering the period from January 2000 to December 2024. The search strategy utilised Boolean operators combining key terms: (“polycystic ovary syndrome” OR “PCOS”) AND (“bile acid” OR “bile acid metabolism”) AND (“gut microbiota” OR “intestinal microbiome”) AND (“FXR” OR “TGR5” OR “bile acid receptors”). The inclusion criteria encompassed peer-reviewed original research articles, clinical trials, mechanistic studies (including those using animal models), and authoritative reviews, all published in English. Articles were excluded from consideration if they did not explicitly address the GM, BAs, or the pathogenesis of PCOS. Furthermore, exclusions were applied to editorials, conference abstracts that lacked comprehensive data, and studies published before 2000, except for seminal references. We subsequently identified the literature encompassed in this review through the examination of titles, abstracts, and full texts. This targeted methodology facilitated the identification and integration of pivotal studies, which constitute the foundational evidence for the proposed GM-BAs-PCOS axis discussed in this paper.

## 2 Interactions between BAs and gut microbiota

**Figure 1:**
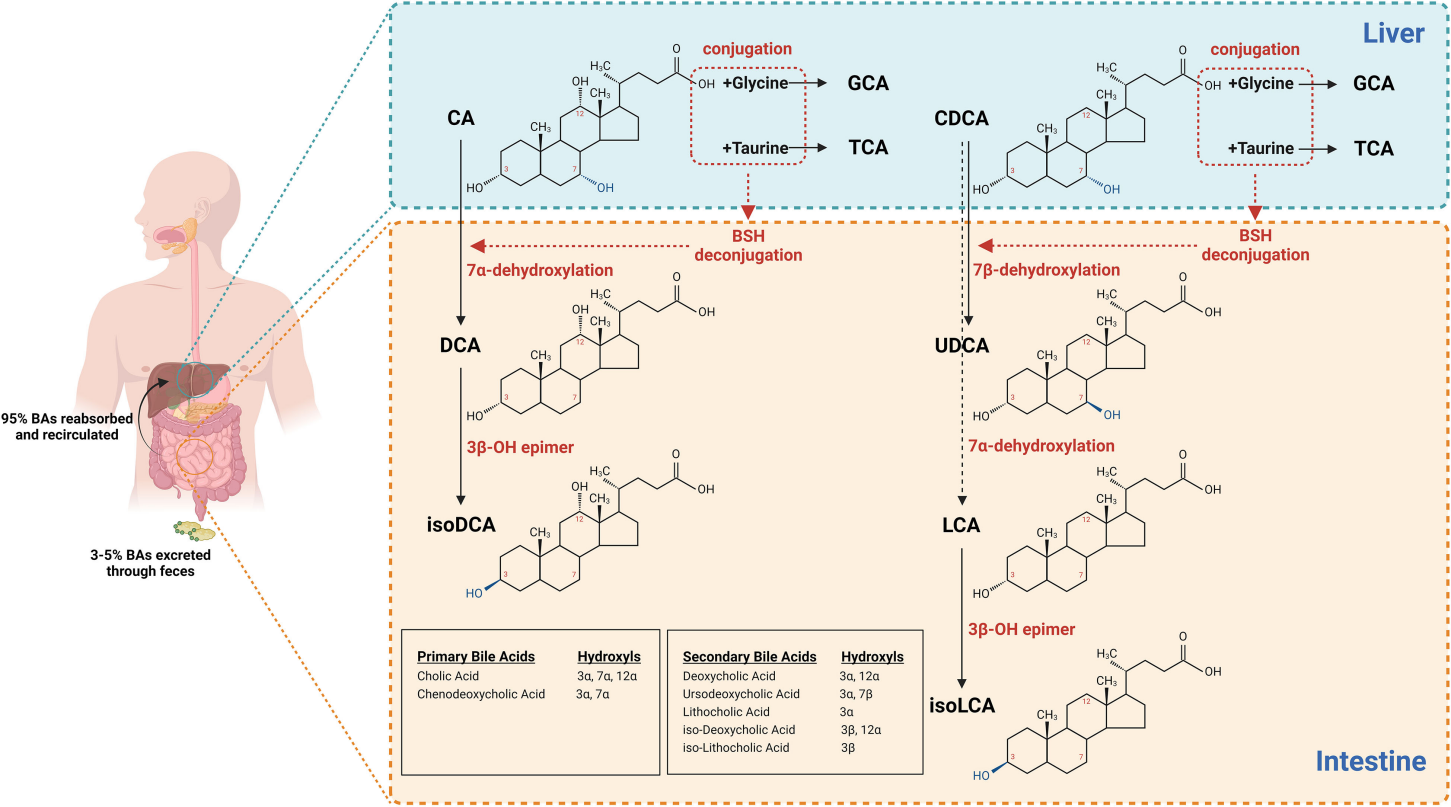
Chemical transformations of BAs by gut microbiota. Chemical structures of primary bile acids CA and CDCA. Primary BAs are conjugated with either glycine or taurine by an amide bond (peptide bond) before secretion. Chemical structures of secondary bile acids DCA, LCA, and UDCA modified by GM via 7α-dihydroxylation/7β-dihydroxylation. Chemical structures of secondary BAs isoDCA and isoLCA 3β-OH epimerised of DCA and LCA.

The gastrointestinal microbiome is the most prolific natural ecosystem, comprising at least several thousand bacterial species. Approximately 99.1% of the functional genes within our genetic repertoire are estimated to be derived from the microbiome (Zhu et al., 2010; Pasolli et al., 2019). The microbiome can modulate the host’s physiological functions through its metabolites. GM produce secondary BAs via the biotransformation of host-derived primary BAs (de Aguiar Vallim et al., 2013; Ramírez-Pérez et al., 2017; Collins et al., 2023). The constituents of circulating BAs include (1) Primary BAs, which are synthesised from cholesterol within hepatocytes; (2) Secondary BAs, which are generated through bacterial modification of primary BAs in the distal intestine (Hofmann and Hagey, 2008; Ridlon et al., 2016). Notably, nearly 100% of BAs in the large intestine undergo dehydrogenation by broad-spectrum bacteria (Devlin and Fischbach, 2015; Wahlström et al., 2016). BAs are predominantly processed via the highly efficient enterohepatic circulation *in vivo*, with hepatocytes and intestinal cells actively facilitating BAs transport to maintain the daily circulation of the BAs pool (Stellaard et al., 1984). 95% of BAs are reabsorbed and recirculated, while the remaining 5% enter the terminal ileum, cecum, and colon, serving as substrates for bacterial biotransformation (Begley et al., 2005). These unabsorbed BAs are converted into secondary BAs through three distinct biological pathways: C24-amide hydrolysis, facilitated by the expression of bile salt hydrolase (BSH); dehydrogenation, executed by a wide range of intestinal anaerobic bacteria; and 7α-dehydroxylation reactions, which are restricted to a limited group of intestinal anaerobes constituting a minor fraction of the overall intestinal flora (Lin et al., 2023). This series of reactions converts the glycine and taurine conjugates of the primary BAs, cholic acid (CA) and chenodeoxycholic acid (CDCA), into the secondary BAs deoxycholic acid (DCA), lithocholic acid (LCA), and ursodeoxycholic acid (UDCA). Furthermore, DCA and LCA can undergo microbial modification to form other secondary BAs, such as iso-deoxycholic acid (isoDCA) and iso-lithocholic acid (isoLCA). These derivatives may possess cytotoxic and/or mutagenic properties, potentially contributing to oxidative stress, membrane damage, and colonic carcinogenesis. This transformation process is considered the most significant quantitative process in the intestinal microbial community (Ridlon et al., 2016; Režen et al., 2022) (**Figure 1**).

BAs serve as amphipathic biological detergents and potent signalling molecules, functioning as both a physical/chemical defence mechanism and a key environmental factor that systematically regulates gut function. They are crucial for directly interacting with both commensal and pathogenic gut microorganisms *in vivo* (Copple and Li, 2016). Firstly, amphiphilicity enables BAs to exhibit direct antibacterial properties, disrupting bacterial cell membranes and altering intracellular macromolecular structures, thereby inhibiting bacterial proliferation. The selection mechanism regulates GM by promoting a relative reduction in the proportion of susceptible species in bile-tolerant bacteria, which can also affect microbiota diversity (Bustos et al., 2018; Winston and Theriot, 2020). A high-fat diet can stimulate the liver to produce more taurocholic acid (TCA). TCA not only specifically promotes the growth of sulfite-reducing bacteria, but also significantly reduces the abundance of *Firmicutes*, indicating how BAs components regulate specific microbial communities (Devkota et al., 2012). Supporting evidence for this function is derived from models of biliary tract obstruction, which demonstrate notable proliferation and translocation of gut microflora (Watanabe et al., 2017; Santiago et al., 2019). Secondly, BAs regulate intestinal barrier function. GM metabolises primary BAs into more hydrophobic secondary BAs, such as DCA and LCA. Secondary BAs have the potential to compromise the integrity of the intestinal epithelium by disrupting tight junctions, thereby increasing paracellular permeability and contributing to the manifestation of a “leaky gut” phenotype (Stenman et al., 2013; Sinha et al., 2020). Unique BAs, especially UDCA, in addition to BA receptor agonists (those that activate FXR and TGR5), not only benefit mucosal repair, but also strengthen tight junctions, thus alleviating inflammation. This activation leads to the upregulation of host defence genes, including those encoding antimicrobial peptides, thereby inhibiting excessive bacterial proliferation in the ileum and playing a crucial role in safeguarding the distal small intestine from bacterial invasion, thereby protecting barrier function (Inagaki et al., 2006; Gadaleta et al., 2011a; Anderson and Gayer, 2021). Thirdly, BAs regulate gastrointestinal physiological functions through receptor-mediated pathways, during which activation of FXR and TGR5 in intestinal endocrine cells, intestinal neurons, and immune cells not only benefits intestinal motility and secretion, but also provides relevant support for immune regulation. For example, TGR5 activation in enterochromaffin cells stimulates serotonin release, which affects gut motility, while in L-cells it promotes glucagon-like peptide-1 (GLP-1) secretion, linking BAs signalling to metabolic and secretory responses (Alemi et al., 2013; Ní Dhonnabháín et al., 2021). BAs regulate intestinal function through a triple action mechanism: firstly, they systematically alter nutrient supply through antibacterial selection, thereby shaping microbial community structure and related abundance; second is to regulate intestinal epithelial cell permeability directly through cytotoxic effects or receptor-mediated barrier integrity maintenance; In addition, it can directly affect secretion motor and sensory functions by activating receptors expressed throughout intestinal mucosa and neural architecture. Thus, BAs represent a significant challenge to the survival and colonisation of GM.

Reciprocally, the disruption of the intestinal microbial community results in the dysregulation of BAs metabolism. Intestinal microorganisms possess three distinct microbial hydroxysteroid dehydrogenases (HSDHs): 3α-, 7α-, and 12α-HSDH, which facilitate the specific oxidation of hydroxyl groups and the exopolymerization of BAs (Sinha et al., 2020; Shalon et al., 2023). Research has demonstrated that oral antibiotics, prebiotics, and probiotics can modify both the structure and function of GM and alter the BAs pool (Qi et al., 2019; Wu et al., 2019). This phenomenon can be attributed to the impact of antibiotic interventions on the intestinal microecosystem, which is associated with a reduction in 7α-dehydroxylation activity, thereby altering BAs metabolism (Aguirre et al., 2021). On the contrary, certain Gram-positive bacteria possessing 7α-dehydroxylation capability, such as Clostridium, a member of the Firmicutes phylum, can facilitate the production of secondary BAs (Marion et al., 2019). Consequently, the microbial community likely exerts a substantial impact on nutrient absorption and BAs toxicity, contingent upon its specific capacity for BAs transformation, thereby influencing the host’s metabolic response. Recent evidence indicates that administering probiotics for disease prevention and treatment can modify the composition of GM and BAs, resulting in health benefits such as reduced cholesterol levels, weight loss, and enhanced insulin sensitivity (Sivamaruthi et al., 2020; Bonfili et al., 2022). Therefore, it is evident that the composition of BAs is influenced by and dependent on the presence of GM.

In summary, BAs can alter the community structure of intestinal microorganisms through various direct and indirect mechanisms. Conversely, intestinal microorganisms can modulate the BAs pool, thereby forming and shaping the interconnected GM-BAs-host axis. Alterations in any component of this axis can lead to dysfunction in the other components. Numerous diseases are influenced by this linkage axis, which also represents a novel therapeutic target. Advancing our understanding of this axis could significantly contribute to the development of precision medicine.

## 3 BAs specific structural formulas and their acidic environment value

Bile is a yellow-green aqueous solution comprising a mixture of organic and inorganic compounds, which becomes acidified following the removal of water and electrolytes through Na+/H+ exchange (Wang et al., 2019). BAs are hydroxysteroids synthesised via a multienzyme pathway from cholesterol in the liver. Their molecular structure features a perhydrocyclopentanophenanthrene steroid nucleus, consisting of three fused six-membered carbon rings and a fourth five-membered carbon ring, along with a short side chain terminating at a carboxyl group. These BAs can be conjugated to glycine or taurine through the formation of an amide (peptide) bond (Horáčková et al., 2018). (**Figure 1**.) At physiological PH, unconjugated BAs exhibit limited solubility (Russell, 2003; Sun et al., 2024). In contrast, the conjugated form of BAs, being a strong acid, undergoes complete ionisation at intestinal PH, thereby enhancing the aqueous solubility of BAs in acidic environments. This characteristic is essential to their function as absorption modulators, as they can markedly enhance the solubility and bioavailability of poorly soluble drugs. BAs serve as solubilising agents and permeation enhancers, promoting drug absorption through diverse mechanisms (Pavlović et al., 2018). Concurrently, the lipophilic properties of BAs confer antimicrobial activity, with bacterial cell membranes as primary targets (Tremblay et al., 2017; Bustos et al., 2018). BAs can interact with cell membranes, phospholipids, and proteins, disrupting cellular homeostasis and consequently exerting cytotoxic effects. Numerous studies have demonstrated that BAs exert broad-spectrum detrimental effects on membrane integrity, potentially by enhancing membrane permeability, which can lead to cell death (Fuentes-Broto et al., 2009; Perez and Briz, 2009). Elevated concentrations of bile salts have been shown to rapidly solubilise membrane lipids, causing the dissociation of integral membrane proteins (Coreta-Gomes et al., 2015; Forooqi Motlaq et al., 2021). Electron microscopy has revealed cellular atrophy and cavitation following exposure to BAs, while enzyme assays have confirmed leakage of intracellular substances. These findings collectively suggest that BAs significantly alter the integrity and permeability of cell membranes. The extent of cell damage is closely related to the structure and hydrophobic nature of BAs (Stenman et al., 2013). Conjugated BAs, being strong acids, are typically fully ionised and remain in the outer hemileaflet of the bilayer at physiological PH. Unconjugated BAs, on the other hand, passively move through the lipid bilayer into cells, with those containing dihydroxy groups and greater hydrophobicity having higher flip-flop efficiency and posing more risk to membrane integrity (Bustos et al., 2018). In addition to causing membrane damage, BAs can destabilise macromolecules by disrupting RNA secondary structure, leading to DNA damage and protein misfolding (Bustos et al., 2018). These properties endow BAs with potent antibacterial capabilities, thereby playing a crucial role in the body’s physicochemical defence system.

Obviously, the presence of BAs as environmental signals poses an extreme challenge to the survival of intestinal microorganisms. Consequently, the tolerance of pathogens and symbiotic bacteria to BAs is of great significance for their survival and subsequent colonisation of the gastrointestinal tract.

## 4 BAs and intestinal epithelial barrier integrity in PCOS

The integrity of the intestinal mucosa is maintained through a dynamic equilibrium among cell proliferation, differentiation, and apoptosis, enabling it to consistently resist the invasion of harmful substances, such as bacteria, toxins, and antigens, within the intestinal environment. This balance is crucial for the mucosa’s roles in digestion, absorption, and endocrine function (Sánchez de Medina et al., 2014). Furthermore, the intestinal epithelial barrier, regulated by tight-junction proteins, serves a protective function by safeguarding the organism from the toxic contents of the intestinal lumen (Marchelletta et al., 2021). Primary BAs are metabolised by intestinal microbiota into more hydrophobic secondary BAs, DCA and LCA, which exhibit cytotoxic properties by damaging bacterial membranes and altering intracellular macromolecular structures (Cremers et al., 2014). *In vitro* and non-PCOS model studies indicate that these secondary BAs can enhance epithelial permeability to macromolecular probes in a concentration-dependent manner (Sinha et al., 2020). They also disrupt barrier function in colonic biopsy tissue from healthy individuals or those without PCOS, through mechanisms involving epidermal growth factors and other pathways. This disruption facilitates the absorption of antigens and bacteria, allowing luminal contents such as endotoxins and bacterial fermentation products to enter the circulation, thereby triggering and maintaining an inflammatory state of the intestinal mucosa (Wang et al., 2020). However, iso-DCA and iso-LCA, produced by modification of intestinal microorganisms, reduce the bactericidal effect of BAs (Devlin and Fischbach, 2015). In contrast, *in vitro* experiments utilising the paracellular permeability of Caco-2 cells - a widely accepted *in vitro* model for studying human intestinal permeability - demonstrated that identical concentrations of primary BAs, CA, and CDCA exert minimal or no effect on paracellular permeability. This finding suggests that microbial transformation by intestinal bacteria enhances the biological activity of BAs within the gut (Scheithauer et al., 2016). Only microbial populations that can tolerate high concentrations of BAs can survive in the gut. In non-PCOS animal models, Lotta K. Stenman et al. investigated the potential pathogenic role of BAs in barrier dysfunction by incubating jejunal and colonic segments from mice with DCA. They confirmed that DCA impairs intestinal barrier function. They also analysed the effect of DCA on cyclooxygenase-2 (COX-2) content in the colon using Western blot, and the results suggest that DCA has detrimental effects on intestinal epithelial integrity and tissue destruction (Stenman et al., 2013; Ruiz-Malagón et al., 2022). On the contrary, studies not specific to PCOS show that UDCA, recognised as the most hydrophilic and least toxic BAs in the human body, demonstrates a direct anti-secretory effect on colonic epithelial cells and facilitates epithelial wound healing, thereby safeguarding the intestinal barrier (Mroz et al., 2018; Goossens and Bailly, 2019).

**Figure 2:**
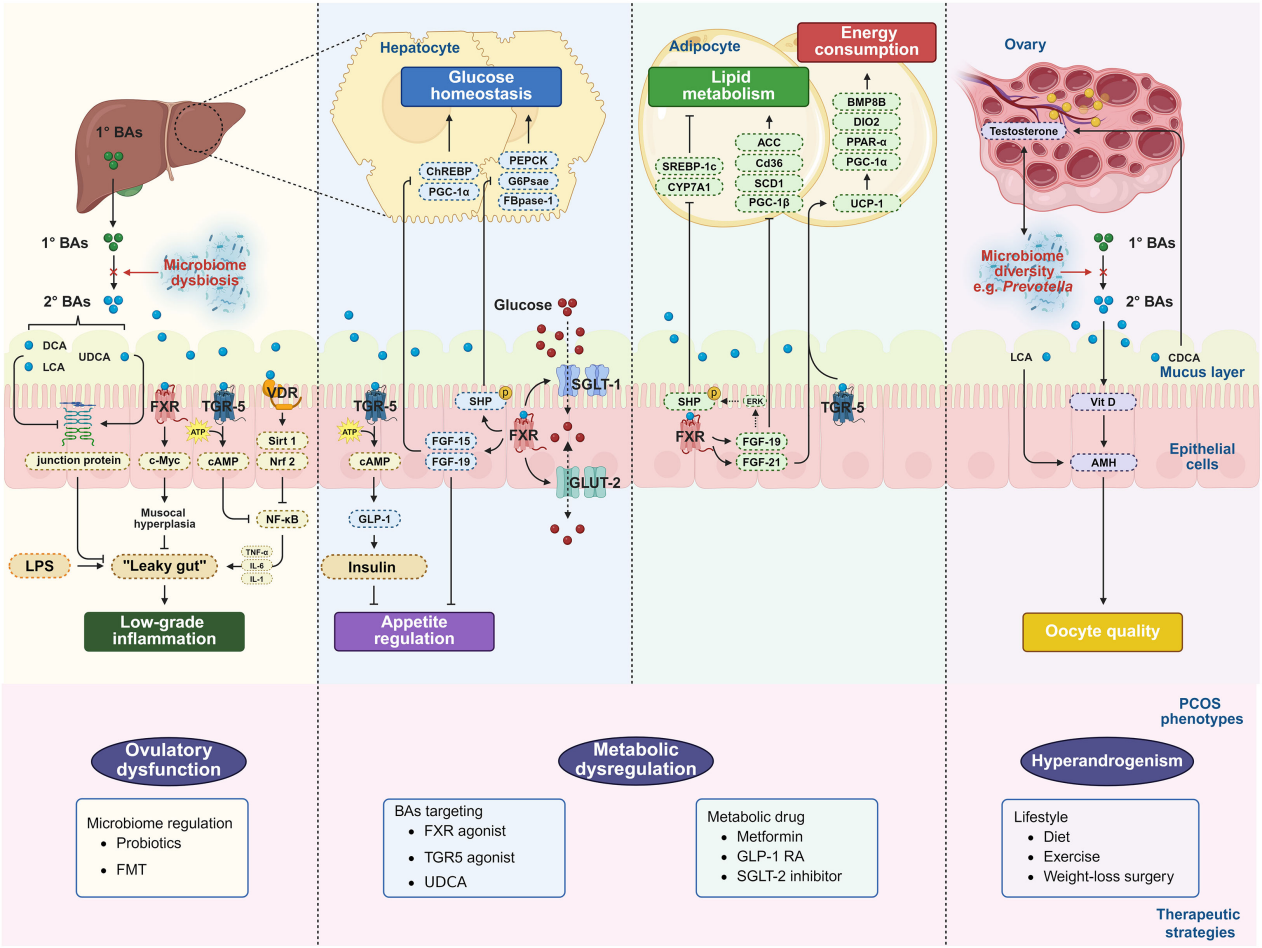
Integrated schematic of the GM–BAs axis in the pathogenesis of PCOS. Primary BAs are released from the liver into the gut and converted into secondary BAs, which activate FXR and TGR5. (1) FXR induces the expression of SHP, FGF-19, FGF-15 and FGF-21 to regulate glucose and lipid metabolism. (2) Also, FXR elevates the abundance of SGLT1 and GLUT2 to regulate the uptake and transport of glucose. (3) TGR5 induces enterocytes to release GLP-1 to promote insulin release and reduce blood glucose levels. (4) TGR5 also induces UCP-1 to promote fat browning and energy consumption. (5) Activation of TGR5 inhibits transcriptional activity of NF-κB, thereby inhibiting the expression of pro-inflammatory cytokines.

An increasing body of evidence indicates that BAs are involved not only in digestion and absorption but also in signal transduction and the regulation of cell growth and survival via various receptor pathways. BAs can activate FXR, and this activation of the BAs-FXR pathway can lead to intestinal mucosal proliferation, inhibition of bacterial growth, and prevention of intestinal barrier disruption. Thus, FXR mediates intestinal protection through BAs (Inagaki et al., 2006; Distrutti et al., 2015). Studies based on a non-PCOS intestinal injury model have demonstrated that taurodeoxycholic acid (TDCA) can facilitate the proliferation of intestinal mucosa via FXR-mediated upregulation of c-Myc protein expression and confer protection against intestinal barrier damage induced by lipopolysaccharide (LPS) (Zahiri et al., 2011). In addition, BAs exert non-genomic effects through the activation of TGR5, a member of the G protein-coupled receptor superfamily, prominently expressed in the ileum and colon, and functions as a cell-surface receptor activated by BAs (Alemi et al., 2013). In target cells, the TGR5 signalling pathway, activated by secondary BAs, such as LCA, stimulates the conversion of adenosine triphosphate (ATP) to cyclic adenosine monophosphate (cAMP). This process exerts anti-inflammatory effects by inhibiting nuclear factor kappa B (NF-κB)-mediated production of proinflammatory cytokines and preserving the integrity of the intestinal barrier (Tough et al., 2020; Zhao et al., 2023). On the contrary, based on TGR5 knockout animal models, not specific to PCOS, the absence of TGR5 may result in structural disruption of colonic tight junction molecules, heightened intestinal permeability, and an increased response to barrier-disrupting agents, this indicates that TGR5 plays a crucial role in the regulation of intestinal homeostasis and supports the hypothesis that TGR5 provides regulatory signals to intestinal epithelial cells (Sorrentino et al., 2020) (**Figure 2**). Additionally, it is well-established that the VDR, when activated by 1,25-dihydroxyvitamin D3, is essential for maintaining the integrity of the intestinal mucosal barrier by upregulating tight junction proteins (Meckel et al., 2016; Zhou et al., 2024). *In vitro* studies utilising Caco-2 cells have demonstrated that LCA exerts a significant protective effect against tumour necrosis factor-α (TNF-α)-induced impairment of intestinal barrier function via VDR. This protective effect may be mediated by inhibition of the NF-κB signalling pathway and by activation of the silent information regulator 1 (SIRT1) and the nuclear factor erythroid2-related factor 2 (Nrf2) pathway (Yao et al., 2019).

The “leaky gut” hypothesis posits that compromised intestinal barrier function leads to heightened antigen exposure, which may initiate a cascade of inflammatory responses, potentially contributing to the pathogenesis of conditions such as inflammatory bowel disease (IBD), irritable bowel syndrome (IBS), and metabolic syndrome (Tilg et al., 2022). Lisa Lindheim et al. proposed that a “leaky gut” allows the LPS produced by Gram-negative bacteria to traverse the intestinal wall into the circulation, linking the resulting endotoxemia to the systemic low-grade inflammation seen in PCOS patients. Supporting this, a metagenomic analysis found that the altered GM in PCOS patients was positively correlated with elevated serum testosterone, LH, and AMH levels, suggesting that dysbiosis may exacerbate endocrine dysfunction by disrupting the barrier and inducing endotoxemia (Chu et al., 2020). They also confirmed that changes in intestinal barrier function were associated with specific endotoxemia markers in PCOS patients, as assessed by 16S rRNA gene amplicon sequencing (Lindheim et al., 2017; Haudum et al., 2020). In non-PCOS animal studies, GM has been shown to affect systemic metabolism by altering the intestinal epithelial barrier, leading to accumulation of bacterial endotoxins (Cani et al., 2008; Thaiss et al., 2018). Based on the findings from non-PCOS model studies, endotoxemia caused by intestinal flora imbalance and intestinal barrier damage can promote PCOS-related IR, HA, and lipid storage by up-regulating inflammatory signals (Tremellen and Pearce, 2012; Gomes et al., 2018). Previously, the relationship between intestinal microbiota and intestinal barrier damage in PCOS patients has been preliminarily studied using PCOS animal models, confirming that faecal microbiota transplantation (FMT) can improve PCOS phenotypes (Quaranta et al., 2019). Given that most mechanistic insights are derived from non-PCOS models, further work in human models is needed to determine whether BAs regulate the intestinal barrier integrity in patients with PCOS via GM.

## 5 BAs-mediated glucose homeostasis in PCOS

FXR and TGR5, two critical regulators of BAs synthesis in the human body that are colocalised in enteroendocrine L cells, have been implicated in the regulation of insulin synthesis, secretion, and sensitivity, as well as in glucose metabolism through a variety of pathways (Jia et al., 2024). Consequently, they have been converted into new therapeutic targets for diabetes (Chávez-Talavera et al., 2017). In models of metabolic diseases without PCOS, BAs activate FXR and induce intestinal expression of fibroblast growth factor 19 (FGF-19) during the postprandial period. Concurrently, they upregulate the expression of genes encoding the inhibitory nuclear receptor small heterodimer partner (SHP). SHP subsequently inhibits gluconeogenesis by suppressing the activities of key enzymes involved in the hepatic gluconeogenesis pathway, such as phosphoenolpyruvate carboxykinase (PEPCK), glucose-6-phosphatase (G6Pase), and fructose 1,6-bisphosphatase-1 (FBPase-1) (Lundåsen et al., 2006; Ma et al., 2006; Kir et al., 2011). Meanwhile, fibroblast growth factor 15 (FGF-15), induced by FXR, deactivates the carbohydrate response element binding protein (ChREBP), which is stimulated by glucose, leading to downregulation of peroxisome proliferator-activated receptor γ co-activator-1α (PGC-1α). This process modulates glycolytic gene expression and suppresses hepatic gluconeogenesis (Caron et al., 2013). The increased presence of Bacteroides was replicated in the PCOS mouse model. The depletion of GM led to improvements in the PCOS phenotype and IR, alongside elevations in relative FXR mRNA levels in the ileum and in serum fibroblast growth factor 15 levels. Bacteroides is a critical microbial biomarker in PCOS and has potential diagnostic value. Gut dysbiosis may contribute to IR. Activation of FXR may have a beneficial rather than adverse effect on glucose metabolism in the context of PCOS (Yang et al., 2021). In non-PCOS animal models, FXR was significantly reduced in BAs-deficient mice, accompanied by obvious IR and hyperglycemia, whereas FGF-19 transgenic mice were resistant to diet-induced obesity and IR (Zhao et al., 2020, 2021). In the small intestine, transepithelial glucose transport is mainly mediated by the sodium-glucose transporter 1 (SGLT1) and the glucose transporter 2 (GLUT2) in intestinal epithelial cells (Wright et al., 2011). SGLT1-and GLUT2-knockout mice showed varying degrees of impaired intestinal glucose absorption and decreased blood glucose levels (Röder et al., 2014; Schmitt et al., 2017). Studies in non-PCOS metabolic models have demonstrated that BAs-activated FXR can not only facilitate glucose uptake in intestinal epithelium by elevating the expression of SGLT1 and GLUT2, but also inhibit transepithelial glucose transport by altering the ratio of their abundance, and effectively control the glucose flux into circulation. Meanwhile, the inhibition is specific for glucose and unrelated to intestinal permeability injury (Zhao et al., 2020). In studies using non-PCOS models, FXR antagonists Z-Gugg and GW4064 were used to elucidate the glucose-regulatory effects of BAs-mediated FXR. It was found that Z-Gugg significantly reversed the inhibitory effect of CDCA on glucose transepithelial transport. At the same time, GW4064 significantly inhibited electrogenic glucose transport and PEPCK- and G6Pase-mediated gluconeogenesis, and improved insulin sensitivity (Zhang et al., 2006). These results indicate that BAs-mediated FXR reduces the transepithelial glucose transport from the lumen to the circulation and plays a role in maintaining the dynamic balance of glucose homeostasis *in vivo*. However, these mechanisms are primarily based on studies in non-PCOS animal or metabolic disease models. Recent studies not specific to PCOS populations have also suggested that FXR is expressed in human pancreatic β-cells and stimulates insulin-related gene transcription, playing an active role in controlling glucose-dependent insulin secretion and regulating peripheral insulin sensitivity (Kong et al., 2024; Yan et al., 2024). (**Figure 2**).

TGR5 has been identified as a BAs-activated membrane receptor, highly expressed in epithelial cells of the gastrointestinal system and exposed to high levels of BAs. The conjugated and free BAs bind and activate TGR5 in the order of TLCA>LCA>DCA>CDCA>CA (Hua et al., 2021; Tveter et al., 2023). GLP-1 is critical for regulating glucose homeostasis, appetite, and the secretion of insulin and glucagon by the pancreas (Gribble and Reimann, 2021). On the one hand, gut microorganisms regulate the TGR5 signal transduction by producing agonists, thus promoting the secretion of GLP-1, leading to the release of glucose-dependent insulin and regulating glucose homeostasis, a mechanism primarily studied in diabetes/obesity models (Sinclair et al., 2018). On the other hand, the specific TGR5 agonist 6α-ethyl-23(S)-methyl-CA(6-EMCA) can activate TGR5 signalling, increase the level of intracellular cAMP, induce colonic L cells to secrete GLP-1, and promote the expression of GLP-1 receptor, accounting for the influence on insulin sensitivity and glucose metabolism, based on non-PCOS metabolic studies (Pathak et al., 2018). In the BD mice model, TGR5 expression and circulating GLP-1 levels were increased, and serum total BAs levels were significantly positively correlated with circulating GLP-1, indicating that BAs signalling triggered GLP-1 release (Pierre et al., 2016). Overexpression of TGR5 in transgenic mice without PCOS observably improved glucose tolerance on a high-fat diet, and this effect was associated with increased GLP-1 secretion and insulin release. Similarly, TGR5 knockout mice with non-PCOS had reduced levels of the BAs pool, along with over-fat accumulation (Maruyama et al., 2006). Moreover, TGR5 can also promote the browning of adipose tissue, induce uncoupling protein-1 (UCP-1), and activate peroxisome proliferator-activated receptor-α (PPAR-α) and PGC-1α to increase mitochondrial oxidative phosphorylation and energy metabolism, thus preventing obesity and diabetes (Zhai et al., 2021; Wang et al., 2022; Li et al., 2023), (**Figure 2**).

Together, these findings from non-PCOS models establish the fundamental roles of FXR and TGR5 in glucose metabolism. Their relevance to PCOS, a condition characterised by high rates of IR and metabolic dysregulation, is an area of active investigation. According to relevant surveys, IR affects about 50%-70% of women with PCOS, leading to many complications, including metabolic syndrome, dyslipidemia, abnormal glucose tolerance, diabetes, etc., and the incidence is higher in obese individuals, especially those with abdominal obesity (Teede et al., 2023). PCOS patients generally have the phenomenon of obesity caused by dietary disorders. A study reported that obese individuals had higher serum total BAs levels than normal-weight individuals, but the response of circulating BAs to food stimulation was inhibited (La Frano et al., 2017). The contribution of the GM-BAs axis to glucose dysregulation in PCOS is likely influenced by BMI status. In high-fat diet-induced obesity and diabetes models, adipose tissue inflammation and more pronounced dysbiosis may lead to increased intestinal permeability and metabolic endotoxemia, which, in turn, exacerbate hepatic and peripheral IR (Cani et al., 2008). In contrast, disturbances in BAs signalling in lean PCOS might stem from more intrinsic defects in microbial community function or BAs receptor sensitivity. In the PCOS cohort containing individuals with lean body types, specific changes were observed in the circulation patterns of BAs profiles, including an increase in conjugated primary BAs levels, revealing a potential BAs-mediated IR mechanism that is independent of obesity (Zhu et al., 2024). This phenotypic heterogeneity implies that therapeutic strategies targeting BAs receptors or downstream effectors, such as GLP-1, may need to be evaluated in the context of patient adiposity. Serum metabolomics analysis of patients with PCOS using liquid chromatography-mass spectrometry (LC-MS) indicated that BAs, as potential biomarkers, are associated with the multiple aetiologies and complex pathogenesis of PCOS (Jia et al., 2019). Whole-genome shotgun sequencing indicated that the gut microbial community structure in PCOS was significantly different from that of healthy controls, and the abundance of the BSH gene, which is mediated by microorganisms, increased, accompanied by decreases in GDCA and TUDCA. Transplantation of faecal microorganisms from PCOS patients into recipient mice can induce a similar PCOS phenotype, including IR and changes in BAs metabolism. Further investigation based on PCOS animal model studies revealed that BAs administration enhanced the expression of TGR5 and cAMP signalling molecules, significantly increased the relative expression levels of UCP-1 and PGC-1α in subcutaneous and brown fat, and reversed ovarian dysfunction and IR in mice with PCOS phenotype (Qi et al., 2019). Similarly, UDCA intervention in letrozole-induced PCOS rat models showed that UDCA significantly reduced the number of cystic and atretic follicles, lowered serum total testosterone levels, and corrected IR (Gozukara et al., 2016). Metformin is currently the preferred choice to correct IR in patients with PCOS. It can inhibit the synthesis and output of liver glycogen, increase the absorption and utilisation of glucose in peripheral tissues, and improve insulin sensitivity. A meta-analysis found that GLP-1 receptor agonists were superior to metformin in improving insulin sensitivity and reducing body mass index (BMI) in women with PCOS, presenting a potential therapeutic opportunity (Cena et al., 2020). It is worth noting that the clinical basis of FXR/TGR5 agonists, FGF-19/FGF-21 agonists, and GLP-1 receptor agonists in the treatment of PCOS is still lacking, and the main support is their related effects on diabetes and obesity, so their application in this disease is still largely speculative or in an early stage of discussion. In addition, based on obesity/diabetes models, GLP-1 receptor agonists can increase browning of white adipose tissue and enhance fibroblast growth factor (FGF-21) expression, thereby reducing visceral fat accumulation (Yadav et al., 2022). Studies in obese patients (not specifically with PCOS) have shown that bariatric surgery significantly improves weight loss and IR, and that FXR and TGR5 signals are positively correlated with increased serum BAs, GLP-1, and FGF-19 levels after bariatric surgery (Münzker et al., 2022). It turns out that BAs’ metabolism was one of the key metabolic pathways affected by changes in GM in individuals with PCOS; BAs-activated intestinal FXR and TGR5 may be effective therapies for the treatment of obesity and glucose metabolism abnormalities in PCOS patients. However, this inference is mainly based on obesity/diabetes model data, and the direct mechanism of PCOS still needs further exploration. GLP-1 may be an important regulatory signal linking the reproductive and metabolic systems, and it is of great significance for preventing long-term complications of PCOS (Yun et al., 2024).

## 6 BAs-mediated lipid metabolism in PCOS

The prevalence of obesity is increasing year by year globally and has now reached epidemic levels. By using multi-omics to systematically compare changes in GM regulation associated with lipid metabolism in PCOS patients, relevant studies not only confirm that the GM-BAs axis still plays a core role during this period, but also reveal its comprehensive impact mechanism on dyslipidemia (Yang et al., 2022). Although there are many reasons for the increase in obesity, one key factor is excessive food intake (Engin, 2024). Consuming a high-fat diet increases microbial abundance and7α-dehydroxylation activity in GM, promoting the conversion of secondary BAs and potentially enhancing fat absorption. The synthesis and transport of BAs and cholesterol are closely related to BMI (Sun et al., 2022). Studies using various techniques have shown that the levels of BAs and cholesterol in morbidly obese individuals are significantly increased, and the levels can be normalised by weight control through calorie restriction or fasting (Straniero et al., 2017). In animal studies, it was found that GM promotes diet-induced obesity in a manner dependent on the BAs receptor FXR in mice (Parséus et al., 2017). FXR regulates lipid and lipoprotein metabolism by acting on hepatic lipogenesis, lipoprotein secretion, and intestinal cholesterol absorption (Valencia-Ortega et al., 2023). Reorganising the GM structure can regulate the BAs-FXR pathway, thereby improving dyslipidemia in rats with PCOS (Duan et al., 2025).

FGF-19 and FGF-21 are members of a gene subfamily with unique properties, the presumed “hormone-like” actions of which can regulate glucose/lipid metabolism and energy homeostasis in multiple target organs (Cicione et al., 2012). In high-fat diet-induced obesity models, FGF-19 and FGF-21 lowered serum glucose, triglyceride (TG), and cholesterol levels, improved insulin sensitivity, and reduced body weight (Würfel et al., 2023). On the one hand, BAs are the centraltranscriptional regulators of FGF-19, and the BAs’ nuclear receptor, FXR, is the primary regulator of FGF-19 (Camilleri and Nurko, 2022). Early research using non-PCOS FGF-19 transgenic mice first revealed that FGF-19 positively regulates glucose, lipid metabolism, and energy homeostasis (Fu et al., 2004). Increased BAs levels in the postprandial intestinal epithelium activate FXR, which in turn induces the secretion and expression of FGF-19 in terminal ileum cells (van Nierop et al., 2019). FGF-19 binding to the FGF receptors can decrease the transcription of a series of genes closely involved in adipogenesis, including acetyl-CoA carboxylase (ACC), Cd36, sterol regulatory element-binding protein-1c (SREBP-1c), stearoyl-CoA desaturase 1 (SCD1), and cytochrome P450 7A1 (CYP7A1), as well as inhibit the expression of lipogenic enzymes by decreasing the expression of peroxisome proliferator-activated receptor γ co-activator-1β (PGC-1β) (Bhatnagar et al., 2009). Meanwhile, FGF-19 can activate extracellular signal-regulated kinase (ERK) signalling in adipocytes and phosphorylate SHP, thereby repressing histone modification of CYP7A1 and inducing steatolysis (Qu et al., 2025). In conclusion, primarily based on studies in non-PCOS models, the FXR-SHP-SREBP-1c/CYP7A1 cascade may play a central role in adipogenesis (Peng et al., 2022; Liu et al., 2024).

On the other hand, in the fasting/starvation state, BAs increase hepatic FGF-21 gene transcription and secretion by activating PPAR-α, which, in cooperation with PPAR-α, regulates gluconeogenesis and fatty acid oxidation in the liver (Fougerat et al., 2022; Ahmadi et al., 2023). During the ingestion state, FGF-21 has insulin-like properties. It can be considered an adipokine that acts as an autocrine fed-state factor in white adipose tissue (WAT) and is up-regulated by peroxisome proliferator-activated receptor-γ (PPAR-γ) to regulate adipocyte function and gene expression (Fisher and Maratos-Flier, 2016). Meanwhile, studies based on metabolic models show that FGF-21, as an effective inducer of UCP-1 in WAT, can cooperate with TGR5 to promote thermogenesis by enhancing PGC-1α activity (Cuevas-Ramos et al., 2019). It has been demonstrated primarily in obesity/metabolic animal models without PCOS that FGF-21 can effectively induce the browning of WAT and increase the expression of the thermogenic factors such as BMP8B, DIO2, PGC-1α, and PPAR-α in adipose tissues, including beige and brown adipose tissue (BAT) (Gao et al., 2014; Mendez-Gutierrez et al., 2024). Moreover, FGF-21 can stimulate adiponectin expression, which plays an important role in maintaining glucose and lipid metabolism balance in the body (Lin et al., 2013). Furthermore, FGF-21 can increase glucose transporter 1 (GLUT1) expression in adipocytes. It can also mediate thermogenesis and energy consumption via the axis of brain adipose tissue, to alter food intake and energy expenditure (Liu et al., 2018). (**Figure 2**).

The dysregulation of lipid metabolism in PCOS cannot be disentangled from adipose tissue biology and distribution, which vary significantly between lean and obese phenotypes. In addition to the thermogenic function of BAT, both WAT and beige adipose tissue are integral to the dysregulation of lipid metabolism observed in PCOS. Dysregulation of adipokine secretion and continuous low-grade inflammation interfere with the function of WAT, which significantly promotes the formation of IR and metabolic syndrome in PCOS patients (Mukherjee et al., 2023). In obese PCOS, excess WAT mass is a major driver of dyslipidemia. Dysbiosis of GM leads to enrichment of hydrophobic secondary BAs, such as DCA, which in turn promotes lipid absorption and adipogenesis (Sun et al., 2022). Although patients with lean PCOS have lower overall fat mass, they often exhibit abnormal abdominal fat distribution and adipocyte dysfunction (Li et al., 2019). Observed BAs disturbances, such as CDCA elevation in individuals, might influence lipid metabolism through different pathways, perhaps directly affecting liver fat and steroid production (Zhu et al., 2024). WAT is transformed into beige adipocytes, during which they utilise FGF-21 and activate TGR5 signaling through BAs and enhance systemic metabolism by increasing energy expenditure (Cuevas-Ramos et al., 2019; Li et al., 2023). In PCOS, adipose tissue can be structurally damaged, leading to reduced beige ability assessment, lipid accumulation, and exacerbation of metabolic dysfunction (Li et al., 2019). TGR5 and FXR, two BAs receptors, systematically regulate lipid production, lipid storage, and inflammatory response in white adipose tissue, thereby maintaining overall metabolic stability and providing protection for body health. This is not only beneficial for metabolic balance, but also for the normal functioning of physiological systems (Zhai et al., 2021; Valencia-Ortega et al., 2023). It is crucial that the GM-BAs-FXR/TGR5 axis imbalance, which is commonly present in PCOS, is also a key factor in evaluating the functional impairment of beige adipose tissue, thus revealing its role as a core upstream node in pathophysiology. Consequently, based on the aforementioned mechanisms derived from non-PCOS models, targeting the BAs-FXR/TGR5-FGF21 axis to enhance white adipose tissue browning and restore adipose tissue homeostasis constitutes a promising therapeutic strategy for addressing lipid metabolic abnormalities in PCOS (Sun et al., 2022).

Obese PCOS patients, as a model of insulin resistance combined with metabolic syndrome, often exhibit changes in circulating FGF-19/FGF-21 levels (Ramanjaneya et al., 2020; Yoost et al., 2022). In the study of metformin’s effects on fibroblast growth factors (FGFs), it was found that metformin can effectively increase bile flow, regulate BAs metabolism, induce the expression of FGF-19/FGF-21, change the blood glucose and lipid spectrum of rats, and improve glycolipid metabolism (Wang et al., 2017; Pedersen et al., 2024). Another study suggested decreased glycocholic acid levels in PCOS were closely associated with fat absorption dysfunction (Yang et al., 2021). Therefore, based on the above research, which includes both associations observed in PCOS populations and mechanistic insights primarily derived from non-PCOS models, it is inferred that regulation of FGF-19/FGF-21 expression by GM-mediated BAs has potential therapeutic value for abnormal lipid metabolism in obese PCOS patients, and further clinically controlled studies are urgently needed to analyse the role of BAs in lipid spectrum metabolism and energy homeostasis in PCOS.

## 7 BAs-mediated appetite regulation in PCOS

Intestinal hormones that cause satiety after a meal include cholecystokinin (CCK), GLP-1, peptide YY (PPY), oxyntomodulin (OXM), pancreatic polypeptide (PP), among others. In human studies of obese individuals without PCOS, these hormones have been shown to work in concert and provide a strong anorexigenic signal (Farhadipour and Depoortere, 2021). Evidence from *in vitro* and animal model studies without PCOS suggests that BAs stimulate enterocyte secretion of intestinal hormones, such as GLP-1 and PYY, which regulate blood glucose and appetite (Christiansen et al., 2019). This effect depends on the activation of TGR5 receptors on the enteroendocrine cells, with the relative agonist potency as follows: LCA > DCA > CDCA > CA (Kuhre et al., 2018). Supporting this, a human study in obese patients without PCOS by Hansen M et al. demonstrated that chenodeoxycholic acid increased insulin-to-glucose ratio, delayed gastric emptying, decreased desire for food, and inhibited weight gain in individuals on a limited diet, proposing a physiological role for BAs in appetite control (Hansen et al., 2016). Subsequent investigations using animal models of obesity and diabetes without PCOS have shown that, in intestinal endocrine L-cells with high receptor abundance TGR5, activated by BAs and dietary agents, stimulates GLP-1 secretion. On the one hand, GLP-1 exerts its prominent incretin effect, enhancing postprandial glucose disposal. On the other hand, it can regulate gastrointestinal motility, inhibit gastric emptying, and thus produce a sense of satiety after meals (Wang et al., 2023). Human studies in lean and obese individuals have confirmed that GLP-1 increases satiety perception and reduces ad libitum energy intake (Jin et al., 2024). Intriguingly, intraperitoneal administration of a GLP-1 receptor antagonist attenuated the anorexigenic effects of GLP-1, suggesting that a peripheral GLP-1 receptor may mediate the suppressive effects on food intake in normal physiology (Jepsen et al., 2021). (**Figure 2**.) Relevant research in rodent models of obesity without PCOS has also indicated that BAs may exert a “direct” postprandial anorexigenic effect via the FXR-mediated downstream signal of FGF-19. The peripheral and central administration of FGF-19 can reduce food intake and body weight in rodents (Marcelin et al., 2014; Ryan et al., 2014). Another study showed that BAs affect food intake by regulating fat-dependent GPR119 bioactivity in the distal intestine, thereby influencing gastric emptying and satiation (Higuchi et al., 2020). In addition, leptin is a satiety-regulating peptide that signals eating behaviour; secondary BAs are essential for the effective absorption of dietary lipids in the intestine and may trigger the release of regulatory peptides (Bensignor et al., 2024). Studies have confirmed that the composition and concentration of BAs in the enterohepatic circulation regulate the leptin gene expression in adipocytes, thereby inhibiting appetite and reducing body weight (Lawler et al., 2020).

Clinical research in women with PCOS has shown a significantly increased risk of hyperglycemia, hyperlipidemia, hypertension, and cardiovascular and cerebrovascular diseases. This is associated with the dysregulated appetite control mechanisms and the obesity caused by impaired satiety responses (Stefanaki et al., 2024). Obesity, as an important outcome of disrupted appetite regulation, serves not only as a clinical manifestation of PCOS but also as a pivotal factor that accelerates the progression of the disease (Ibáñez and de Zegher, 2023). As mentioned earlier, the molecular mechanism by which BAs regulate appetite through the TGR5/GLP-1 and FXR/FGF-19 pathway is mainly constructed in non-PCOS systems and covers the physiological and pathological characteristics of human obesity and various metabolic diseases. Direct evidence from animal PCOS models or human studies in women with PCOS specifically elucidating these appetite-regulatory pathways remains limited. However, given the shared metabolic features between PCOS and the conditions studied—particularly obesity, IR, and dysregulated satiety signalling—it is plausible to extrapolate that similar BAs-mediated mechanisms may contribute to appetite dysregulation in PCOS. This extrapolation forms a rational basis for investigating the GM-BAs-PCOS axis. Disorder in PCOS can affect BAs metabolism, thereby interfering with appetite-regulation mechanisms and causing disease-related high appetite and weight gain. By targeting BAs signalling pathways, such as TGR5 or FXR, it is expected to form a potential treatment plan for PCOS associated with obesity, which is not only beneficial for improving metabolic abnormalities, but also for controlling weight gain. It is necessary to confirm, through comparison of PCOS-specific patterns and clinical trials, studies that elucidate the association between BAs metabolism and appetite hormones in PCOS population, revealing potential connections and transforming the working mechanism into an efficient evaluation of therapeutic intervention strategies.

## 8 BAs-mediated sexual hormones change in PCOS

The GM exhibits sexual dimorphism. Females generally possess greater microbial diversity, whereas males show a higher abundance of *Prevotella*, a genus positively correlated with testosterone levels and negatively associated with oestradiol concentration (Falony et al., 2016; Haro et al., 2016; Chen et al., 2017). Patients with PCOS often have significantly elevated levels of *Prevotella* in the gut, leading to HA (Li et al., 2023). In clinical practice, GM diversity of those patients is still negatively correlated with serum testosterone, indicating that HA continues to reshape GM structure. This mechanism is also used to explain pathological associations, which is beneficial not only for understanding pathogenesis, but also for guiding clinical interventions. In addition, they systematically reveal this bidirectional regulatory relationship (Chu et al., 2020). A regression analysis demonstrated a significant correlation between gut microbial diversity and HA, as measured by serum testosterone levels and hirsutism, suggesting that high-androgen composition, with microbial diversity diminishing as testosterone levels rise (Torres et al., 2018). These observations imply a bidirectional interaction between sex hormones and the GM. The causal relationship and mutual influence between sex hormones and GM still need to be fully elucidated. Multi-omics analysis shows a significant correlation between GM metabolites and HA in PCOS patients and systematically reveals metabolic pathways to disrupt steroid hormone homeostasis through biological dysregulation. This can also provide a theoretical basis for related mechanisms (Yang et al., 2022). It is worth noting that changes in GM diversity and elevated testosterone levels jointly impair gut permeability, which can lead to IR involvement in the progression of PCOS (Li et al., 2019, 2023).

Recent studies are elucidating the molecular mechanisms that connect BAs with steroidogenesis. The specific BAs, such as CA, activate FXR in ovarian theca cells, directly inhibits the expression of steroidogenic enzymes, such as cytochrome P450 17A1 (CYP17A1) and cytochrome P450 7B1 (CYP7B1), Causing a reduction in androgen synthesis (Zhu et al., 2025). Conversely, an excess of androgens can influence hepatic BAs synthesis. It upregulates enzymes like cytochrome P450 8B1 (CYP8B1), favouring the production of 12α-hydroxylated BAs (e.g., CA), which are associated with IR (Haeusler et al., 2013; Petrov et al., 2020). Under the action of androgens, the transformation of BAs pool can reconstruct GM, forming a potential feedback mechanism: androgens→changes in BAs metabolism→dysbiosis of microbiota→endocrine metabolic dysfunction. The metabolic profile of PCOS includes an increase in primary BAs such as CDCA, and a decrease in secondary BAs such as LCA (Zhang et al., 2019). Subgroup analysis reveals an obvious increase in concentrations of CA and CDCA in PCOS with HA. This not only indicates its role in the pathological process of HA, but also helps to elucidate relevant mechanisms (Yu et al., 2023; Zhu et al., 2024). In a randomised controlled trial targeting women with PCOS in China, serum total BAs levels of high androgen hormone patients were significantly higher than those of the normal control group. This systematic study not only confirms the association between androgen excess and abnormal BAs homeostasis, but also provides important evidence for clinical evaluation, which helps to more accurately explore its pathological mechanism and requires in-depth analysis with relevant indicators (Cai et al., 2019). The correlation between key points is complex, and when encountering function of BMI, obesity can autonomously increase testosterone to evaluate changes in related GM (Zhang et al., 2022). Nonetheless, the correlation between distinct bile acid profiles, such as increased CDCA levels, and HA remains evident even in lean PCOS cohorts. This indicates that disruption of steroid synthesis induced by BAs constitutes a core pathological mechanism that remains consistent across different BMI groups and further worsens in the obese state. In addition, secondary BAs and other metabolitesresulting from GM dysbiosis can decrease vitamin D levels, thereby influencing AMH levels in follicular fluid and thereby diminishing oocyte quality (Li et al., 2023). Supplementation with LCA has been shown to mitigate the decline of oocyte quality. Consequently, the combined assessment of CDCA, LCA, and testosterone offers greater predictive value for PCOS than the measurement of testosterone alone. In support of the association between BAs metabolism and HA, the administration of Nicotinamide Mononucleotide in mice with PCOS mitigated hormonal dysregulation. This suggests that interventions aimed at normalising BAs profiles may also address androgen excess (Ren et al., 2024).

It is worth noting that the correlation between the characteristics of different BAs substances and diseases such as PCOS or HA mainly comes from early studies, particularly cross-sectional analyses and case-control studies. Although research consistently indicates modifications in BAs composition, with an increase in 12α-hydroxylated BAs (Haeusler et al., 2013) and an increase in primary BAs in PCOS (Yu et al., 2023), these observational results still lack of systematic validation in clinical diagnosis, effective evaluation, or treatment strategies. Significant individual differences, dietary preferences, BMI, and comparison of drug intervention effects in the BAs pool pose significant challenges, lack of standardised metabolomics planning schemes exacerbates the dilemma. Distribution of body fat (which often exhibits atypical patterns in PCOS patients) not only autonomously affects BAs metabolism, but also significantly correlates with microbiome diversity. Identifying the unique properties of BAs substances in PCOS faces multiple obstacles (Zhang et al., 2022). Although there are biomarkers worth noting in BAs research pathway, their clinical applicability is still insufficient, Therefore, it is urgent to carry out systematic in-depth analysis to construct causal chain validation critical points, in order to evaluate actual effectiveness of BAs in diagnosis and prognosis prediction of PCOS, This is not only beneficial for clinical practice, but also provides support for subsequent research.

## 9 BAs-mediated inflammatory response in PCOS

A range of chronic inflammatory disorders, including obesity, type 2 diabetes, and non-alcoholic steatohepatitis, are linked to alterations in BAs metabolism and pool composition (Thibaut and Bindels, 2022; Li and Chiang, 2024). Hydrophobic BAs are potent inflammatory agents that can injure the liver, intestine, and other tissues, whereas hydrophilic BAs exhibit potent anti-inflammatory properties. Studies conducted in models of metabolic and inflammatory diseases have established that BAs-activated receptors, such as FXR and TGR5, suppress inflammation in macrophages, intestines, and the liver via inhibiting NF-κB nuclear translocation and counteracting NF-κB-dependent pro-inflammatory cytokines (Perino et al., 2014). Research in animal models without PCOS indicates that FXR, in particular, plays a regulatory role in modulating the intestinal mucosal inflammatory response induced by GM (Jia et al., 2019). LPS produced by GM can stimulate inflammatory cells via activating NF-κB (Qu et al., 2021). In models of chronic intestinal inflammation, the overexpression of the p50 and p65 subunits of NF-κB can directly suppress FXR activity, resulting in diminished inhibition of intestinal inflammation and, consequently, facilitating its progression (Gadaleta et al., 2011b). By using cell culture methods to explore the mechanism of action of inflammatory biological patterns, BAs attenuate NF-κB transcriptional activity, thereby inhibiting the expression of pro-inflammatory cytokines. Furthermore, similar non-PCOS models demonstrate that BAs can also elevate cAMP levels via TGR5 activation, thereby inhibiting the production of LPS-induced pro-inflammatory mediators, including TNF-α, IL-1, and IL-6 (Sinha et al., 2020). (**Figure 2**.) Berberine, a bioactive compound extracted from medicinal plants, such as *Coptis chinensis*, is clinically used to treat gastrointestinal inflammation. Preclinical investigations using general inflammation models suggest that berberine may exert anti-inflammatory effects through modulation of the GM-BAs axis. Specifically, it exerts a selective inhibitory effect on bacterial BSH activity, leading to an elevated concentration of tauro-conjugated BAs, including TCA (Shu et al., 2021). The resultant increase in tauro-conjugated BAs activates intestinal FXR signalling, which in turn enhances the expression of mucosal defense genes and the synthesis of antimicrobial peptides (Gadaleta et al., 2011a; Shu et al., 2021). From the perspective of its mechanism of action, berberine activates FXR by regulating BAs, inhibits nuclear translocation of NF-κB, and effectively blocks transcription process of pro-inflammatory factors, including TNF-α and IL-6 (Chen et al., 2025). Experimental data based on PCOS rodent models showed that berberine administration can regulate intestinal permeability, reduce systemic LPS levels, and decrease expression of colitis biomarkers such as NF-κB p65 and COX-2 (Shen et al., 2021a, 2021b). These findings in PCOS models suggest that berberine may alleviate low-grade inflammation and intestinal barrier dysfunction in PCOS via the GM-BAs-FXR axis. However, this inference combines mechanisms from non-PCOS models with phenotypic improvements in PCOS models, and clinical validation is warranted. Interestingly, in the cohort of patients with insulin resistance (not specific to PCOS), the proportion of 12α-hydroxylated BAs was found to be upregulated (Haeusler et al., 2013). Extrapolating from this finding in non-PCOS insulin resistance, it is hypothesised that chronic low-grade inflammation may be involved in the earlier pathological processes of PCOS, potentially triggering IR and HA, thereby contributing to the progression of PCOS and elevating the risk of long-term complications, such as obesity, T2DM, and cardiovascular disease. The hypothesis of “ the intestinal barrier-endotoxemia-inflammatory mechanism” of PCOS was proposed (Qi et al., 2021). Supporting this, studies suggest that GM disruption in individuals with PCOS increases intestinal mucosal permeability, contributing to the development of enteric endotoxemia. Emerging evidence further highlights the interplay between intestinal oxidative stress and microbial dysbiosis in driving this inflammatory cascade. For instance, intervention with Tempol, a superoxide dismutase mimetic, ameliorated PCOS phenotypes in rats by attenuating intestinal oxidative stress, restoring GM balance (increasing *Ruminococcus_1* and decreasing *Ruminococcus_2*, *Staphylococcus*, among others), and modulating serum metabolites, including bile acids and stachyose. By employing antioxidant interventions to restore the GM-metabolite axis, ovarian function was enhanced, and glucose tolerance improved. This underscores the significant roles that oxidative stress, biological disorders, and metabolic issues play in the inflammation associated with PCOS (Li et al., 2021).

Metagenomic analyses have demonstrated an elevation in pro-inflammatory genera, including *Escherichia* and *Shigella*, within the GM of patients with PCOS (Chu et al., 2020). This increase may facilitate mucosal adhesion and invasion, consequently contributing to epithelial dysfunction and systemic inflammation. It has been observed through human studies in women with PCOS that the abundance of *Fusobacterium, Escherichia, Bacteroides*, *and Prevotella* species in the intestine of PCOS is increased, which enhances the virulence of commensal bacteria by promoting mucosal attachment, invasion, and intracellular persistence, which subsequently leads to epithelial dysfunction and increases barrier permeability. These changes culminate in deleterious inflammatory responses within the host (Thackray, 2019). Similarly, studies on a pro-inflammatory gut environment have consistently shown that inflammatory mediators such as IL-6, IL-8, and TNF-α are systematically elevated in PCOS patients, reflecting the prevalence of chronic low-grade inflammation (Zhai et al., 2021; Mukherjee et al., 2023). Disentangling the source of this inflammation is complicated by the high prevalence of obesity in PCOS. In obese models, inflammation likely arises from a combination of hypertrophied adipose tissue (which releases adipokines and free fatty acids) and obesity-associated gut dysbiosis, leading to endotoxemia (Cani et al., 2008). In individuals with lean PCOS, the inflammatory are more specifically reflected in their intrinsic dysregulation, BAs-mediated intestinal barrier disruption, and partial ovarian inflammation (Mukherjee et al., 2023). Research integrating observations from non-PCOS barrier injury models with the pathophysiology of PCOS indicates a potential connection: excessive BAs can compromise the integrity of the intestinal mucosal barrier, trigger the release of pro-inflammatory factors, and cause chronic ovarian inflammation, ultimately impairing ovarian function. An imbalance in intestinal microbiota results in abnormal BAs metabolism, which induces inflammation by compromising the intestinal barrier, thereby exacerbating IR, HA, and ovarian dysfunction (Ommati et al., 2019; Lin et al., 2021; Fu et al., 2023). Providing more direct, albeit preliminary, mechanistic insight, a study in a murine PCOS model observed that BAs can facilitate the synthesis of IL-22 via the GATA binding protein 3 (GATA3) and signal transducer and activator of transcription 3 (STAT3) signalling pathway, thereby ameliorating the clinical phenotype of PCOS (Wang et al., 2021; Gao et al., 2022). Overall, the evidence points to a complex interaction: although the detailed molecular mechanisms of BAs signalling through FXR/TGR5 have been elucidated in non-PCOS models, and dysbiosis and inflammation have been observed in patients with PCOS, direct causal evidence linking specific BAs-mediated pathways to inflammation in PCOS remains limited. The existing data, however, strongly support the hypothesis of a crosstalk among the intestinal microbiota, BAs, and persistent inflammatory signals, all of which are intricately associated with the occurrence and progression of PCOS. Therefore, further investigation aimed explicitly at elucidating the role of GM-BAs signalling pathways in inflammatory manifestations of PCOS is crucial. Future studies stratifying by BMI are needed to determine if the GM-BAs axis contributes to inflammation through distinct mechanisms or microbial signatures in lean versus obese PCOS phenotypes. Such research would bridge the gap between established mechanisms in related conditions and the specific pathology of PCOS, potentially contributing to the development of novel therapeutic strategies.

Collectively, the studies discussed above highlight the potential of targeting the GM–BAs axis to ameliorate various facets of PCOS pathology. To provide a structured overview of key interventional and mechanistic evidence supporting this approach, relevant preclinical and clinical studies are summarised (**Table 2**).

**Table 2:** Summary of interventional and mechanistic studies relevant to the BAs-PCOS axis.

Study (Year)	Model type	Intervention	Main BAs-related outcomes	PCOS / metabolic outcomes	Relevance for PCOS	Limitations
Zhang et al. (2006)	C57BL/6J, Lepr^db/db^ and Lepr^db/?^, KK-A(y) mice	FXR agonist (GW4064) Overexpress FXR (Ad-FXR-VP16)	Implied role in BAs homeostasis via FXR	↓ Hyperglycemia, hyperlipidemia, hepatic steatosis ↑ Insulin sensitivity, glycogen synthesis ↓ Gluconeogenic genes (G6Pase, PEPCK)	- FXR activation improves glucose/lipid metabolism - Relevant to PCOS-associated IR and dyslipidemia	- No direct PCOS model - Clinical translation unclear - Mechanisms in PCOS not addressed
Gozukara et al. (2016)	Letrozole-induced PCOS rat model	UDCA (150 mg/kg/d) vs. metformin	UDCA improved ovarian morphology (↓ cystic & atretic follicles, ↑ antral follicles)	↓ Total testosterone ↓ Insulin levels ↔ lipids, glucose, HOMA-IR, estrogens	- First to show UDCA improves ovarian histology and androgen levels in PCOS - Potential for UDCA as a therapeutic agent in PCOS	- Rat model may not fully replicate human PCOS - Anesthesia confounds glucose results - No mechanistic insight into UDCA provided
Wang et al. (2017)	HFD/streptozotocin-induced T_2_DM rats	Metformin (500 mg/kg/d, 12 weeks)	↓ TBAs and TC ↑ Bile flow	Improved FBG, insulin sensitivity, TG, LDL-C ↑ FGF21 ↓ FGF19	- Demonstrates modulation of BAs and FGF19/21 may be part of metformin's mechanism in T2DM - Relevant to PCOS metabolic features	- No direct PCOS model - Not proven causal mechanism for PCOS - Mechanisms not fully elucidated
Pathak et al. (2018)	Wild-type C57BL/6J and leptin receptor-deficient diabetic mice Lepr^db/db^ FXR^-/-^ mice and TGR5^-/-^ mice	FXR agonist (fexaramine)	↑ TLCA, ↑ LCA ↑ BAs hydrophobicity ↑ LCA-produce bacteria (*Acetatifactor, Bacteroides*)	↑ Glucose tolerance, insulin sensitivity, GLP-1 ↑ Adipose tissue browning ↓ Adiposity and hepatic lipids	- FXR/TGR5/GLP-1 improve metabolic dysfunction - Relevant to PCOS (IR, obesity, dyslipidemia)	- Not tested in a PCOS model - Antibiotic-dependent effects - Clinical translatability unclear
Younossi et al. (2019)	Human phase 3 RCT (NASH patients)	Obeticholic acid	Improved liver histology (fibrosis) Increased ALP (on-target effect) Lipid modulation (transient LDL increase)	Improved fibrosis stage ↓ ALT/AST, weight Modest HbA1c increase in diabetics (transient)	- FXR agonism improve metabolic liver disease and IR - Relevant to PCOS-associated NAFLD/NASH	- No clinical outcomes data yet - Biopsy-dependent population - Lipid/glycemic effects require monitoring
Qi et al. (2019)	PCOS women (cohorts) DHEA-treated & PAMH-treated mice	FMT from PCOS women Oral gavage of *Bacteroides vulgatus* IL-22 or GDCA administration	↓ GDCA & TUDCA in PCOS ↑ *bsh* gene in *B. vulgatus* GDCA/TUDCA induce IL-22 via GATA3/TGR5	↑ IR, ovarian dysfunction, infertility, inflammation ↓ Adipose tissue browning, Improved by IL-22/GDCA	- Establishes a direct gut microbiota-BAs-IL22 axis in PCOS pathogenesis - Identifies IL22 as a potential therapeutic target	- Cohort limited to Chinese population - Mouse BAs conjugation differs from human - Causal role of B. vulgatus requires further validation
Zhao et al. (2021)	db/db mice (BKS.Cg-Dock7^m +/+^ Lepr^db^/J) C57BL/6 mice HFD/streptozotocin-induced T_2_DM mice	FXR antagonist (Mebhydrolin) (15/30 mg/kg/d, 5 weeks)	FXR antagonism implied affect BAs signaling	↓ FBG, HbA1c, OGTT, G6Pase, PEPCK ↑ p-GSK3β, glycogen Via FXR/miR-22-3p/PI3K/AKT pathways	- FXR antagonism improves glucose homeostasis and insulin sensitivity - Relevant to PCOS metabolic dysfunction	- No direct PCOS model - Long-term safety and clinical efficacy unknown - Off- target effects possible (H1 receptor)
Li et al. (2021)	DHEA-induced PCOS rats	Tempol (SOD mimetic)	Restored serum bile acids	Improved ovarian function ↓ Testosterone ↑ Glucose tolerance	- Links antioxidant therapy to PCOS via gut-BAs-metabolite crosstalk - highlight intestinal redox state as therapeutic target	- BAs mechanism unclear - Causal link weak - Clinical relevance unknown
Shen et al. (2021a)	DHEA-induced PCOS rat model	BBR (150 mg/kg/d, 6 weeks)	↓ p-PI3K/PI3K, p-AKT/AKT, TNF-α, IL-1/6, caspase-3 ↓ Inflammatory via TLR4/LYN/NF-κB ↓ Ovarian granulosa cell apoptosis	↓ HOMA-IR, FBG, T, body weight Improved ovarian morphology	- Anti-inflammatory & Anti-apoptotic - Improve IR & HA via PI3K/AKT/NF-κB pathway	- Steroidogenic enzymese - Strous cycle & fertility not studied - Cell-type-specific apoptosis unclear
Münzker et al. (2022)	Diet-induced obesity rats/mice	RYGB	↑ TCBAs, ↓ BSH Activation of FXR/TGR5	↑ Glucose tolerance, insulin sensitivity ↓ Reduced adiposity Activated BAT thermogenesis	- BAs-microbiota axis improves metabolic dysregulation and IR-core PCOS traits	- Rodent-human BAs differences - Clinical applicability unconfirmed
Gao et al. (2022)	DHT-induced PCOS rats	Troxerutin; S3I-201	↑ GDCA, TUDCA Correlated with IL-22↑	Improved ovarian morphology ↓ HA ↓ IR ↑ IL-22, activated IL-22R1/STAT3 pathway	- Troxerutin as an IL22 enhancer targeting - Gut-BAs-IL22-STAT3 axis for IR in PCOS	- Rodent model only - Human relevance unknown - Causal link indirect
Li et al. (2023)	HFD-induced obese mice Intestine-specific TGR5 knockout mice	Red ginseng extracts (60/120/240 mg/kg/d, 6 weeks)	↑ TBAs and specific TCBAs in serum/ileum/WAT ↑ ASBT membrane localization via TGR5/cAMP	↓ Body weight, adiposity, hepatic steatosis ↑ Glucose tolerance, insulin sensitivity, GLP-1 ↑ Lipolysis and energy expenditure	- TGR5 activation improves obesity and IR - Relevant to PCOS features (IR, obesity, dyslipidemia)	- No direct PCOS model - WAT-specific TGR5 knockout not verified - Translational relevance to human PCOS unclear
Gan et al. (2023)	Human (obese PCOS patients)	Exenatide+metformin vs. metformin (2 mg/week s.c.+500 mg tid, 2 weeks)	COM: ↑ CDCA, DA3G MF: ↑ DA3G	Both groups: ↓ BMI, ↓ TT, ↑ HDL-c, ↑ ApoA1 COM group: ↓ HbA1c, ↓ FINS, ↓ HOMA-IR MF group: ↔ glucose parameters	- Combined therapy modulates BAs profile (↑ CDCA) and enriches BAs and HPO-axis related pathways - Multi-target metabolic and endocrine regulation superior to metformin alone	- Short duration - Inferred BAs-receptor mechanisms - Obese cohort only - No placebo control
Yun et al. (2024)	Female C57BL/6J mice	Bv, Bv-Δbsh, Bv-ΔspeA, agmatine, liraglutide, DFMA	Bv-Δbsh still induced PCOS-like phenotype without altering BAs profile Agmatine as FXR agonist independent of BAs	Impaired estrous cycles, polycystic ovaries ↑ HA, IR ↓ GLP-1 secretion Liraglutide/DFMA improve phenotypes	Agmatine-FXR-GLP-1 as a novel, BAs-independent pathway in PCOS pathogenesis - ADC inhibition (DFMA) and GLP-1R agonism as potential therapies	- Difficult to quantify relative contributions of bsh vs. speA pathways - Translation to humans unclear - Clinical efficacy of ADC inhibitors untested
Ren et al. (2024)	Letrozole-induced PCOS mouse model	Nicotinamide mononucleotide (a critical precursor of NAD+)	↓ Total serum bile acids NMN partially restored BAs levels Altered bile acids synthesis pathways	NMN improved ovarian morphology ↓ Androgens Did not restore cycles or fully correct metabolism	- NMN ameliorates HA and BAs dysregulation - Links aromatase inhibition to BAs metabolism - Highlights liver-gut axis role in PCOS	- Single time point - No gut microbiota analysis - NMN incomplete efficacy
Qu et al. (2025)	DHEA+HFD-induced PCOS mouse model H_2_O_2_-induced OS in KGN cells	FGF19 overexpression Si-NRF2 ERK inhibitor	↑ FGF15/FGF19 & FGFR4 ↔ FXR, CYP7a1, CYP8b1 ↓ PRDX5 in proteomics (BAs metabolism pathway)	↑ T, BAX, NRF2, HO1 Impaired glucose tolerance ↓ p-ERK/ERK, BCL2 Altered follicular morphology & estrous cycle	- FGF19 upregulation in PCOS - Regulates granulosa cell viability & apoptosis via FGFR4-ERK-NRF2 axis - Links OS, BAs metabolism & hormonal dysregulation	- Small human sample size - Proteomics limited to follicular fluid - In vivo BAs metabolism not fully characterized - Direct causal role of FGF19 in PCOS not validated
Duan et al. (2025)	Letrozole-induced PCOS rats	SGD oral gavage (25 g/kg/d, 2 weeks)	↑UDCA, altered primary/secondary BA ratio Modulated BAs synthesis genes (CYP7a1, CYP27a1, FXR, SHP)	Improved estrous cycles, ovarian histology, E_2_ ↓ Body/ovarian weight ↓ T, TG, T-CHO, LDL-C	- Ameliorates dyslipidemia via gut microbiota (↑Akkermansia) and BAs/FXR pathway modulation	- Correlation-based - Lack protein validation and bacterial causality - Human relevance unknown

↑ indicate increase, ↓ indicate decrease; ↔ indicate no difference;

ALP, alkaline phosphatase; ApoA1, lipoprotein A1; BAT, brown adipose tissue; BBR, berberine; BSH, bile-salt hydrolase; Bv, B. vulgatus; cAMP, cyclic adenosine monophosphate; CDCA, chemodeoxycholic acid sulfate; COM, exenatide combined with metformin group; CYP27a1, cytochrome P450 27a1; CYP7a1, cytochrome P450 7a1; CYP8b1, cytochrome P450 8b1; DA3G, deoxycholic acid 3-glucuronide; DFMA, difluoromethylarginine; DHEA, dehydroepiandrosterone; E2, oestradiol; ERK, extracellular signal-regulated kinase; FBG, fasting blood glucose; FGF 15, fibroblast growth factor 15; FGF 19, fibroblast growth factor 19; FGFR4, fibroblast growth factor-4 receptor; FINS, fasting insulin; FMT, fecal microbiota transplant; FXR, farnesoid X receptor; G6Pase, glucose-6-phosphatase; GATA3, GATA binding protein 3; GDCA, glycodeoxycholic acid; GLP-1, glucagon-like peptide-1; HA, hyperandrogenism; HbA1c, hemoglobin A1c; HDL-c, high density lipoprotein cholesterol; HFD, high-fat diet; HO1, heme oxygenase 1; HPO, hypothalamic-pituitary-gonadal axis; HOMA-IR, homeostasis model assessment-insulin resistant; IL-22, interleukin-22; LDL-C, low density lipoprotein-cholesterol; MF, metformin group; NAFLD, non-alcoholic fatty liver disease; NASH, non-alcoholic steatohepatitis; NRF2, nuclear factor erythroid 2-related factor 2; OGTT, oral glucose tolerance test; OS, oxidative stress; PEPCK, phosphoenolpyruvate carboxykinase; p-GSK3β, phosphorylated glycogen synthase kinase-3β; PRDX5, peroxiredoxin 5; RYGB, roux-en-Y gastric bypass; SGD, Shaoyao-Gancao Decoction; SHP, small heterodimer partner; T, testosterone; TBAs, total bile acids; TC, total cholesterol; TCBAs, taurine-conjugated bile acids; T-CHO, total cholesterol; TG, triglyceride; TGR5, G protein-coupled bile acid receptor; TT, total testosterone; TUDCA, tauroursodeoxycholic acid; UDCA, Ursodeoxycholic acid; WAT, white adipose tissue.

## 10 Conclusions and future perspectives

Drawing from the evidence compiled in **Table 2**, it is apparent that modulating BAs signalling or the GM offers therapeutic potential for PCOS. In the subsequent section, we synthesise these findings to present comprehensive conclusions and propose directions for future research.

GM, as the largest micro-ecosystem within the human body, is crucial for sustaining physiological functions. This ecosystem exhibits distinct metabolic regulatory activities and exerts a broad spectrum of biological effects, including nutrient absorption, material metabolism, inflammatory response, and barrier function. Numerous studies have demonstrated that GM significantly influences the female reproductive endocrine system through regulating BAs metabolism. An imbalanced GM can result in multiple adverse consequences, such as metabolic disorders, chronic low-grade inflammation, and immune dysfunction. It is crucial to synthesise and emphasise that BAs function as potent pleiotropic signalling molecules, exerting direct regulatory effects on the metabolic, endocrine, and reproductive axes beyond their mediation through the GM. BAs primarily via receptors, such as FXR and TGR5, directly modulate core PCOS pathologies. At the metabolic level, postprandial BAs directly activate intestinal FXR, promote FGF19 release, and thereby inhibit hepatic gluconeogenesis. At the same time, when TGR5 is activated, it can rapidly promote GLP-1 secretion, which is not only beneficial for improving insulin sensitivity, but also for generating satiety, providing dual protection for blood glucose regulation (Kuhre et al., 2018; Perino and Schoonjans, 2022). From an endocrine perspective, specific BAs profiles, notably elevated CDCA levels, show a direct association with HA in clinical PCOS cohorts, suggesting a direct interface with steroidogenic pathways (Yu et al., 2023; Zhu et al., 2024). In the reproductive field, secondary BAs, such as LCA, can directly regulate ovarian microenvironment-related functions by activating ovarian signaling channels, such as VDR and GATA3, which is not only beneficial for relieving inflammation but also for improving oocyte levels (Qi et al., 2019; Li et al., 2023). Concurrently, BAs directly attenuate systemic low-grade inflammation by repressing NF-κB-dependent cytokine production via FXR and TGR5 activation in immune cells (Thibaut and Bindels, 2022; Li and Chiang, 2024). Therefore, the pathogenesis of PCOS is influenced by a dual-action model of BAs: one orchestrated through the GM and another executed through their direct endocrine and paracrine signalling on host tissues.

The following conclusions can be drawn from the literature review: 1) The GM modulates the composition of the BAs pool by biotransforming primary BAs derived from the liver. Conversely, BAs influence the community structure of GM through a variety of direct or indirect mechanisms. 2) As environmental signals, BAs present significant challenges to the colonisation of intestinal microorganisms due to their acidic environment and antibacterial properties. 3) Dysbiosis of GM results in a compromised intestinal epithelial barrier in PCOS, and the various forms of BAs transformed by gut microorganisms exert differential effects on intestinal epithelial integrity. It is hypothesised that BAs may influence the integrity of the intestinal barrier in PCOS by interacting with GM. 4) Receptors activated by BAs, such as FXR and TGR5, are implicated in glucose metabolism in PCOS. The GM is also thought to play an important regulatory role in the expression of BAs receptors. 5) The GM regulates lipid metabolism in patients with PCOS in a BAs receptor-dependent manner and influences body weight by modulating appetite-regulating factors, suggesting potential therapeutic implications for obesity in PCOS. 6) The characteristic changes in the BAs profile of PCOS patients are mainly CDCA, and LCA, and are related to testosterone and AMH levels. The combination of CDCA, LCA and testosterone has important clinical value in the prediction of PCOS. 7) There exists a crosstalk among the GM, BAs, and persistent inflammatory signals, which is closely related to the low-grade inflammatory state characteristic of the ovarian microenvironment in PCOS.

The association between gut dysbiosis and PCOS phenotype is emphasised in clinical metagenomics, with a focus on exploring its intrinsic relationship and utilising systematic monitoring and evaluation to reveal the potential therapeutic value of regulating GM. Fusion analysis highlights significant heterogeneity in PCOS, particularly in terms of metabolic phenotype and BMI dimensions. Obesity plays an important role as a key regulator in the GM-BAs-PCOS axis, and it can exacerbate biological disorders, alter bile acid profiles, impair barrier function, and induce systemic inflammation. There is a significant disorder in the properties of BAs in patients with lean PCOS, and axis disorder suggests that this channel is an essential component of the core pathophysiology of PCOS, and this change is not related to obesity. Future research should prioritise systematically dividing participants into lean and obese PCOS phenotypes and setting up appropriate BMI-matched control groups in order to further explore relevant mechanisms. This approach is essential to: 1) Decouple obesity-driven effects from PCOS-intrinsic mechanisms. 2) Identify phenotype-specific microbial and BAs biomarkers for improved diagnosis and subtyping, and 3) Develop and tailor novel therapeutic strategies that target the GM-BA axis—whether through probiotics, prebiotics, BAs sequestrants, FXR/TGR5 modulators, or FMT—based on the patient’s metabolic profile. Acknowledging and systematically addressing this heterogeneity is not merely a methodological refinement but a prerequisite for advancing personalised and effective management of PCOS.

Addressing the specific inquiry regarding clinical translational evidence, several recent studies have begun to explore therapeutic strategies that modulate the GM-BAs axis in PCOS. For instance, Yu et al. demonstrated that patients with PCOS exhibited distinct circulating BAs profiles characterised by elevated primary conjugated BAs, which correlated with HA and IR, thus revealing potential therapeutic value of BAs regulation (Yu et al., 2023). Another study by Qi et al. identified a GM-BAs-IL-22 axis in PCOS. It showed that FMT reduced *Bacteroides vulgatus* abundance in PCOS, and alleviated phenotypes, providing proof-of-concept for therapies targeting the GM to correct BAs metabolism (Qi et al., 2019). Although direct human trials using BAs agonists (e.g., FXR or TGR5 agonists) specifically for PCOS are still limited, emerging evidence from metabolic disorders supports their potential. For example, obeticholic acid (an FXR agonist) has been shown to improve insulin sensitivity and alter GM composition in patients with non-alcoholic steatohepatitis (NASH), suggesting cross-applicability to PCOS (Younossi et al., 2019). Related studies have shown that therapeutic strategies aimed at modifying the BAs pool, including direct intervention, leveraging microbiota regulation, and receptor activatio, is beneficial for optimising intestinal ecological structure, improving core metabolic abnormalities in PCOS, and significantly alleviating endocrine disorders. The basic theories are gradually being applied in clinical settings. However, to move from promising association to established therapy, critical gaps in our current understanding must be addressed. At present, there is a general lack of systematic analysis on intestinal microecology and BAs axis of people with different phenotypes of PCOS, especially comparative study among different subtypes after BMI is stratified, existing mechanism is mostly derived from animal models or data of non PCOS metabolic diseases, and its direct impact on human pathology has not been confirmed. In addition, there is a significant lack of bile acid intervention research (such as FXR/TGR5 agonist) for PCOS, and related efficacy is inferred from obesity or diabetes research, and its specific role in weight loss or blood glucose regulation is still unclear. Therefore, it is urgent to carry out GM-BAs between lean and obese PCOS phenotypes direct comparison of axis mechanisms can identify phenotype specific pathological pathways. In future research, stratified analysis methods should be prioritised, and large-scale population cohort studies should be conducted using multi omics techniques (microbiome, metabolome, bile acid profile). This not only establishes causal relationships, but also screens subtype specific biomarkers. It is necessary to design randomised controlled trials (RCT) to evaluate efficacy of BAs regulators stratified by metabolic phenotype, and to explore how different bacterial populations differentially regulate BAs metabolism, Only in this way can GM-BAs axis be transformed from a theoretical framework into a clinical goal for precise diagnosis and treatment of PCOS.

This overview reveals that the GM-BAs axis constitutes the key pathological mechanism of PCOS, and constructs a systematic framework that integrates metabolic, endocrine, and inflammatory disorders. By focusing on direct interactions between BAs and receptors, as well as microbiota-mediated effects, a new perspective is provided for the development of targeted intervention strategies, including FXR/TGR5 modulators, probiotics, and fecal microbiota transplantation. This strategy is not only beneficial for improving the PCOS phenotype, but also addresses the limitations of traditional research methods.

## Glossary

PCOS: polycystic ovary syndrome
GM: gut microbiota
BAs: bile acids
FXR: farnesoid X receptor
PXR: pregnane X receptor
CAR: constitutive androstane receptor
VDR: vitamin D receptor
TGR5: G protein-coupled bile acid receptor
BSH: bile salt hydrolase
CA: cholic acid
CDCA: chenodeoxycholic acid
DCA: deoxycholic acid
LCA: lithocholic acid
UDCA: ursodeoxycholic acid
TDCA: taurodeoxycholic acid
isoDCA: iso-deoxycholic acid
isoLCA: iso-lithocholic acid
TCA: taurocholic acid
GLP-1: glucagon-like peptide-1
HSDHs: hydroxysteroid dehydrogenases
COX-2: cyclooxygenase-2
LPS: lipopolysaccharide
ATP: adenosine triphosphate
cAMP: cyclic adenosine monophosphate
NF-κB: nuclear factor kappa B
TNF-α: tumour necrosis factor-α
SIRT1: silent information regulator 1
Nrf2: nuclear factor erythroid 2–related factor 2
IR: insulin resistance
HA: hyperandrogenism
IBD: inflammatory bowel disease
IBS: irritable bowel syndrome
FMT: faecal microbiota transplantation
FGF-19: fibroblast growth factor 19
SHP: small heterodimer partner
PEPCK: phosphoenolpyruvate carboxykinase
G6Pase: glucose-6-phosphatase
FBPase-1: fructose 1,6-bisphosphatase-1
FGF-15: fibroblast growth factor 15
ChREBP: carbohydrate response element binding protein
PGC-1α: proliferator-activated receptor γ
SGLT1: sodium–glucose transporter 1
GLUT2: glucose transporter 2
6-EMCA: 6α-ethyl-23(S)-methyl-CA
UCP-1: uncoupling protein-1
PPAR-α: peroxisome proliferator-activated receptor-α
LC-MS: liquid chromatography–mass spectrometry
FGF-21: fibroblast growth factor 21
ACC: acetyl-CoA carboxylase
SREBP-1c: sterol regulatory element-binding protein-1c
SCD1: stearoyl-CoA desaturase 1
CYP7A1: cytochrome P450 7A1
PGC-1β: proliferator-activated receptor γ
ERK: extracellular signal-regulated kinase
WAT: white adipose tissue
PPAR-γ: peroxisome proliferator-activated receptor-γ
BAT: brown adipose tissue
GLUT1: glucose transporter 1
FGFs: fibroblast growth factors
CCK: cholecystokinin
PPY: peptide YY
OXM: oxyntomodulin
PP: pancreatic polypeptide
CYP17A1: cytochrome P450 17A1
CYP7B1: cytochrome P450 7B1
CYP8B1: cytochrome P450 8B1
GATA3: GATA binding protein 3
STAT3: signal transducer and activator of transcription 3
NASH: non-alcoholic steatohepatitis
RCTs: randomised controlled trials
